# Utilizing 3D bioprinted platelet-rich fibrin-based materials to promote the regeneration of oral soft tissue

**DOI:** 10.1093/rb/rbac021

**Published:** 2022-04-13

**Authors:** Ke Yi, Qing Li, Xiaodong Lian, Yapei Wang, Zhihui Tang

**Affiliations:** 1 Second Clinical Division, Peking University School and Hospital of Stomatology, Beijing 100101, China; 2 National Engineering Research Center of Oral Biomaterials and Digital Medical Devices, Beijing 100081, China; 3 Center of Digital Dentistry, Peking University School and Hospital of Stomatology, Beijing 100081, China; 4 Department of Chemistry, Renmin University of China, Beijing 100872, China

**Keywords:** injectable platelet-rich fibrin, 3D bioprinting, oral soft tissue engineering, regenerative medicine

## Abstract

Oral soft tissue defects remain difficult to treat owing to the limited efficacy of available treatment materials. Although the injectable platelet-rich fibrin (i-PRF) is a safe, autologous source of high levels of growth factors that is often employed to promote the regeneration of oral soft tissue, its effectiveness is restrained by difficulties in intraoperative shaping together with the burst-like release of growth factors. We herein sought to develop a bioactive bioink composed of i-PRF, alginate and gelatin capable of promoting the regeneration of the oral soft tissue. This bioink was successfully applied in 3D bioprinting and exhibited its ability to be shaped to individual patient needs. Importantly, we were also able to significantly prolong the duration of multiple growth factors release as compared to that observed for i-PRF. The growth factor bioavailability was further confirmed by the enhanced proliferation and viability of printed gingival fibroblasts. When deployed *in vivo* in nude mice, this bioink was further confirmed to be biocompatible and to drive enhanced angiogenic activity. Together, these data thus confirm the successful production of an i-PRF-containing bioink, which is suitable for the individualized promotion of the regeneration of oral soft tissue.

## Graphical Abstract

**Figure:**
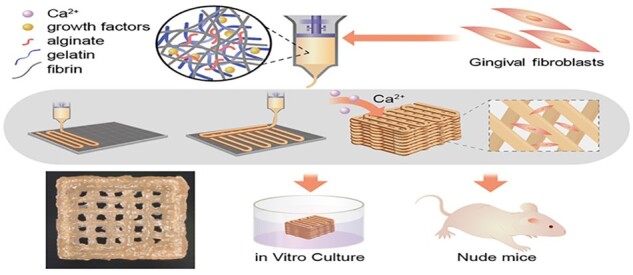

## Introduction

The oral soft tissue is integral to the oral structural and functional integrity. The oral mucosa includes the masticatory mucosa composed of the gingiva and the layer covering the hard palate, as well as the lining mucosa and the specialized mucosa covering the tongue. When oral soft tissue defects arise due to the trauma, infection, cancer or gingival recession, mucosal reconstruction is generally required [1]. A range of different reconstructive autologous tissue grafting techniques can be employed in this context, including subepithelial connective tissue graft and free gingival graft, with the optimal grafting strategy depending on the individual need and the location of the tissue being repaired [2–4]. Such autologous grafting, however, is subject to many limitations and disadvantages including the limited amount of tissue available for grafting, the challenging surgical procedures necessary to complete such grafting, and the reports of long-term postoperative pain and numbness in treated patients [3, 4]. Other approaches aim at reducing graft harvesting-related morbidity have been explored in recent years, including the use of different collagen matrices, soft tissue substitutes and freeze-dried skin allografts [5, 6]. However, these approaches have generally been unsatisfactory and resulted in high rates of shrinkage in the grafted sites [4]. Grafted biological scaffolds must be individually tailored to the specific conformation of the defective tissue undergoing repair, and current histological analyses have revealed marked differences between grafted and natural mucosal tissues [7]. Therefore, clinically there is an urgent need for a self-sourced, easily available, reproducible and biologically active material.

In recent years, blood concentrates products have attracted wide attention in oral and plastic surgery because of their advantages of autologous source, reproducible and high bioactive properties [8–11]. In a pioneering study conducted in 2001, Choukroun *et al*. found that a centrifugation step (2700 rpm, 12 min) was sufficient to process venous blood so as to yield concentrated growth factors in a gel-like fibrin matrix termed platelet-rich fibrin (PRF) [12]. The activation of PRF can trigger the release of key repair-related growth factors including platelet-derived growth factor (PDGF), epidermal growth factor (EGF), vascular endothelial growth factor (VEGF), insulin-like growth factor (IGF), transforming growth factor-β (TGF-β) and fibroblast growth factor (FGF) [8, 9]. PRF is most frequently employed for the treatment of gingival recession [13], extraction sockets and palatal wound closure owing to its safety, autologous sourcing, non-damaging nature and repeatability [14]. Further insight regarding the therapeutic applications of PRF in a range of dental contexts such as endodontics, implantology, sinus lift, socket preservation, bone regeneration, orthodontics and periodontology, consults a recently published review [15]. Despite these promising results, however, in prior reports, PRF has been found to release the majority of growth factors within 7–10 days [16], which limited the applications in oral soft tissue regeneration, as soft tissue healing requires 6–8 weeks for maturation [17]. PRF is also commonly applied in combination with other biomaterials as it cannot be readily shaped to fit oral soft tissue defects.

Further study has revealed that an appropriate low-speed centrifugation can yield the injectable PRF (i-PRF) without any additive anticoagulation [18]. i-PRF samples share the superiority in the increased platelet and growth factor concentrations together with a more compacted 3D fibrin network and thicker protein fibers conducive to capturing platelets and regulating growth factor release. Consistently, i-PRF can more effectively promote the migratory, proliferative and osteogenic activity of gingival fibroblasts and osteoblasts [19, 20]. Injectable features provide operable window time for customized molding.

The use of 3D bioprinting represents an attractive approach to generating soft tissue scaffolds suitable for soft tissue repair owing to the ability of printed scaffolds to conform to the thickness, volume, mechanical properties, shape and functionality of tissues in an individualized manner [21]. Such 3D bioprinting would also enable the use of a digital workflow amenable to producing patient-tailored grafts [22]. Importantly, a range of delivery systems capable of promoting the sustained growth factor delivery and prolonged bioactivity are compatible with 3D bioprinting technologies [23]. Due to the fabrication of interpenetrating polymeric network, alginate/gelatin (AG) bioink has good printing performance and mechanical properties, and is widely used to print mimetic cartilage tissue, blood vessel tissue, skin tissue [24].

**Figure 1.**
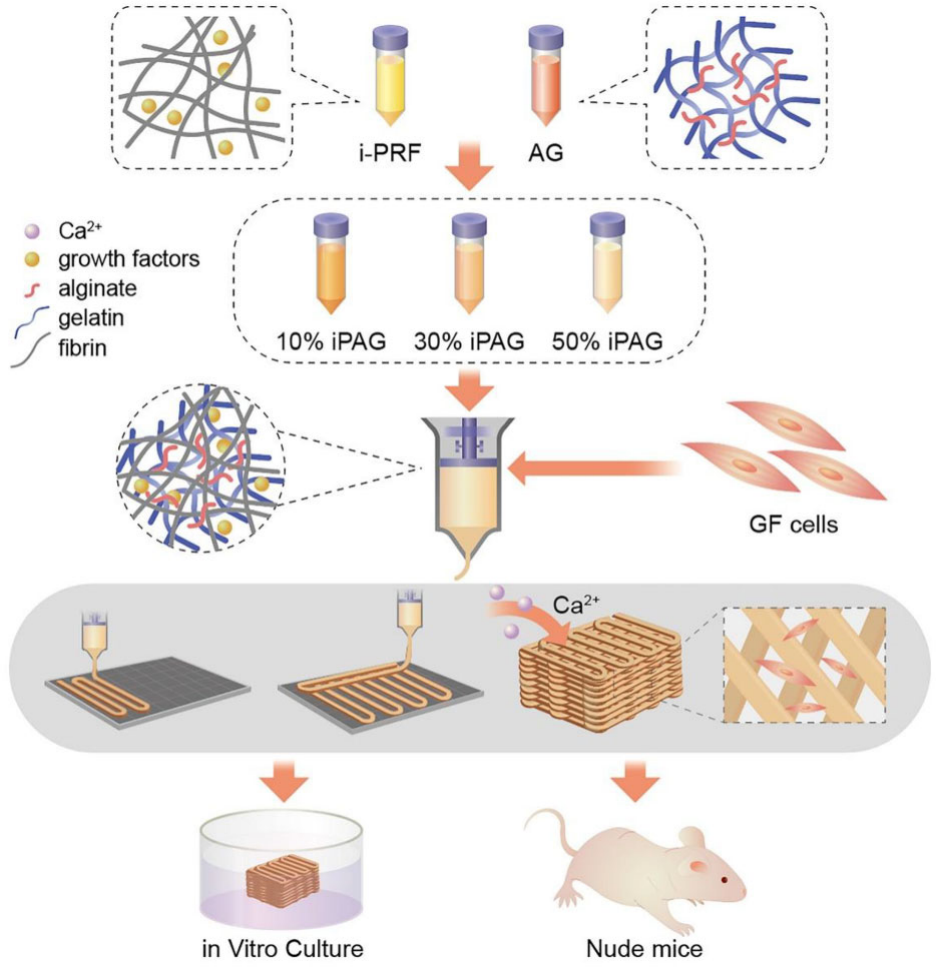
Schematic representation of the study process: from the bioink formulation to the fabrication of 3D bioprinted constructs containing cells, the growth factor release was limited in a compacted fibrin network. *In vitro* cyto-compatibility and *in vivo* biocompatibility of constructs were performed. AG, alginate/gelatin; GF cells, gingival fibroblast cells; iPAG, i-PRF/alginate/gelatin; i-PRF, injectable platelet-rich fibrin

Therefore, in order to improve the release characteristics and personalized molding of PRF in clinical application, In the present study, we prepared an extruded bioink composed of i-PRF, alginate and gelatin for use in the fabrication of constructs amenable to promoting the sustained growth factor release in the context of precision oral soft tissue regenerative therapy (Fig. 1). The physical, mechanical and rheological properties of the prepared bioink were assessed, while its cytocompatibility was evaluated *in vitro* through proliferation and viability assays conducted using human gingival fibroblasts (HGFs). Then, a subcutaneous implantation model using nude mice was employed to evaluate the *in vivo* biocompatibility, angiogenic activity and anti-inflammatory activity of these preparations.

## Materials and methods

### Bioink preparation

#### i-PRF preparation

**Figure 2.**
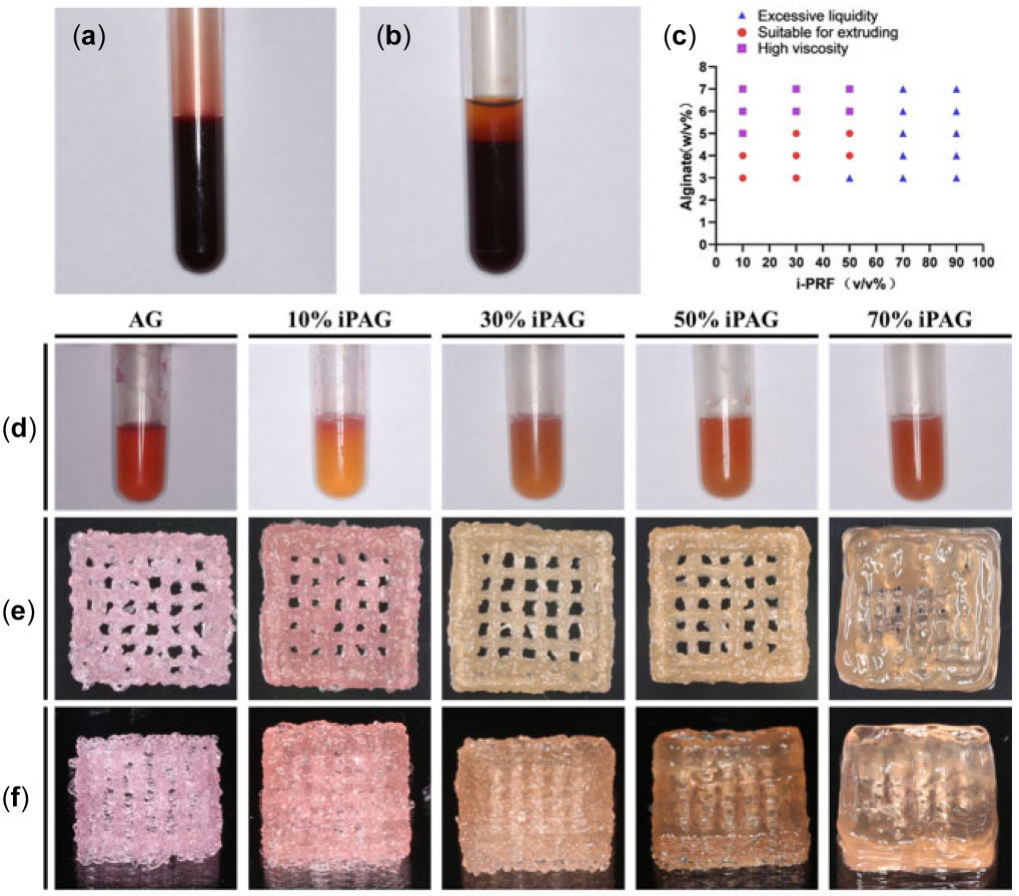
i-PRF preparation process, iPAG bioink formation and extrusion experiments. (**a**) Whole blood. (**b**) After centrifugation. (**c**) Extrusion fluency of iPAG bioinks in different formulations. (**d**) Bioinks with different concentration of i-PRF. (**e**) Front view and (**f**) side view of high resolution photos of the 3D printed constructs

Venous blood samples for the present study were collected from students between 24 and 28 years of age in our laboratory (approved by the Ethics Committee of Peking University School of Stomatology, PKUSSIRB-201950166). All i-PRF preparation was performed as per previously published protocols [18]. Briefly, 6 ml of whole blood (Fig. 2a) in which no anticoagulant had been added was centrifuged (L-400, Lixinjian, Shanghai, China) at room temperature for 3 min at 700 rpm, with the plasma liquid phase then being collected for use as i-PRF (Fig. 2b). Roughly 0.5–1 ml of i-PRF was obtained per 6 ml of venous blood.

#### AG bioink formation

Prior to bioink preparation, gelatin and sodium alginate were sterilized with Co-60 radiation. AG was prepared by mixing 4% (w/v) sodium alginate (Sigma Aldrich) and 8% (w/v) gelatin (Sigma Aldrich) in serum-free α-MEM (Gibco, NY, USA), with samples being agitated at 37°C overnight until fully dissolved. The prepared AG bioink was stored at 4°C.

#### i-PRF/AG bioink preparation

To prepare the i-PRF/AG (iPAG) bioink, the fresh i-PRF was added to the prepared AG at 10%, 30% and 50% volume ratio, with samples then being evenly mixed (Fig. 2d).

#### Material ratio analyses

To explore the optimal bioink formulation amenable to fluid extrusion, a series of experiments were performed using a range of sodium alginate concentrations and i-PRF volume ratios (Fig. 2c). Growth factor concentrations should theoretically be proportional to i-PRF concentrations, but with the i-PRF volume ratio above 50% will result in samples with excessive liquidity, precluding their extrusion to produce filaments. Thus, a maximum 50% (v/v) i-PRF volume was used for bioink preparation. Ultimately, a 4% sodium alginate concentration was selected, with i-PRF volume ratios of 10%, 30% and 50%, yielding the following experimental groups: 4% alginate + 8% gelatin (AG), 10% i-PRF + 90% AG (10% iPAG), 30% i-PRF + 70% AG (30% iPAG) and 50% i-PRF + 50% AG (50% iPAG). Concentrations of different components in these individual preparations are summarized in Table 1.

**Table 1.** Concentrations of alginate, gelatin and i-PRF in each group

	Alginate (w/v)	Gelatin (w/v)	i-PRF (v/v)
AG group	4%	8%	0
10% iPAG group	3.6%	7.2%	10%
30% iPAG group	2.8%	5.6%	30%
50% iPAG group	2%	4%	50%

#### HGF-iPAG bioink preparation

Healthy gingival samples were collected from 20- to 30-year-old patients undergoing crown lengthening in the Periodontology Department of Peking University Hospital of Stomatology, with a tissue block method employed for HGF extraction as published previously [25]. The Ethics Committee of Peking University School of Stomatology approved the study (PKUSSIRB-201950166). Patients provided informed consent for the sample collection. HGFs were grown in proliferation medium consisting of DMEM containing 10% fetal bovine serum and 1% antibiotics in a humidified 5% CO_2_ 37°C incubator. Medium was exchanged every 2–3 days, in which cells were passaged using 0.25% trypsin + EDTA when 80% confluent. All HGFs were used within five passage cycles following isolation. HGF-iPAG bioink was prepared by carefully mixing HGFs (1 × 10^6^ cells/ml) with fresh iPAG bioink.

### iPAG bioink characterization

#### Rheological analyses

A rotational rheometer (Discovery HR2; TA Instruments, Inc., USA) was employed to analyze different bioink preparations, assessing properties including the storage modulus (G′), loss modulus (G″) and composite viscosity thereof. Briefly, experiments were conducted following a 2-min period during which the bioink was allowed to reach equilibrium following the application of 0.6 Pa preshear for 1 min. Individual measurements were made by loading the Peltier stage with the liquid cell-free bioink, with samples being tested a single time prior to their being discarded. Analyses were made at an oscillatory temperature sweep from 35 to 5°C at 1.5°C/min, with a time sweep of up to 10 min and a stress sweep from 0.1 to 1000 at constant frequency and strain values of 1 Hz and 1%, respectively. Strain sweep (0.01–1%) was conducted at a fixed frequency of 1 Hz, while frequency sweep (0.1–100 Hz) was conducted at a fixed strain of 1%. To determine whether there were reductions in the bioink viscosity under shear application over time, rotational steady-state flow was performed at a 0.1–100/s shear rate.

#### Compressive modulus analysis

The compression testing was conducted with a universal testing machine (Instron Model 3342; Illinois Tool Works, Inc., MA, USA) and cross-linked cylindrical test samples (3 mm thick, 5 mm diameter). Room temperature samples were placed between load-bearing sensors with circular metal plates, after which they were gradually compressed at 0.5 mm/min to 80% deformation, with the compressed distance and force values associated therewith being recorded. Strain–stress curves were plotted, and compressive modulus values were then calculated based on the slope of these curves (linear strain range: 0–10%).

#### *In vitro* degradation analyses

To explore the *in vitro* degradability of bioink preparations, 200 μl of individual bioink preparations were added to sterile 37°C PBS following Ca^2+^ cross-linking. At appropriate time points, samples were removed from PBS, freeze-dried and weighed. Initial sample weight (W_1_) and residual mass (W_2_) were then used to establish the degree of degradation at a given time point: residual mass (%) = W_2_/W_1_*100. Samples were analyzed in quadruplicate.

### 3D printed construct fabrication

The fabrication of 3D printed constructs was performed using an extrusion-based 3D bioprinter (Medprin 2.0, China). Initially, the CAD software (MP Bioprint 4.0) was used to model a cube (10 mm × 10 mm × 5 mm). Fresh HGF-iPAG bioink was then loaded into a 1-ml syringe and chilled for 3 min at 4°C to promote gel formation, after which it was installed in the printer. To maximize the accuracy when mimicking biological structures, a needle with a 260-µm inner diameter was selected. The detail parameters used for 3D printing are given in Table 2. After constructs had been printed, sodium alginate cross-linking was achieved by applying calcium chloride for 5 min, after which constructs were transferred into the culture media.

**Table 2.** Parameters for the 3D bioprinting

Parameters	Value
Ambient temperature (°C)	10
Platform temperature (°C)	10
Inner diameter of needle (μm)	260
Fill density (%)	40
Layer thickness (μm)	400
Speed (mm/s)	30

### iPAG 3D printed construct characterization

#### Scanning electron microscopy

The morphological characteristics of the prepared 3D printed cell-free constructs were assessed via scanning electron microscopy (SEM; S-4800; Hitachi High-Technologies Co., Tokyo, Japan). Briefly, 4% paraformaldehyde was used to form cross-linked constructs which there then dehydrated using 70–100% EtOH. Samples were then freeze-dried and sputter-coated with 20 nm-thickness golds.

#### Growth factor release assays

The release of growth factors from cell-free constructs was assessed over a 2-week period during which the Ca^2+^ cross-linked 3D printed samples were incubated in 1 ml of PBS in 24-well plates at 37°C. At selected time points (15 min, 1 h and Days 1, 2, 4, 6, 8, 10, 12, 14), supernatants were collected from each well and stored at −20°C, with fresh PBS then being added. Total growth factor release was quantified using ELISA kits specific for EGF, FGF, VEGF, PDGF-AA, PDGF-AB and TGF-β1 (MultiScience, China) as per provided protocols. Absorbance values for three replicate wells were measured to compute average growth factor concentrations.

A comparable approach was additionally employed to assess growth factor release from i-PRF. Briefly, 200 μl of i-PRF was added per well and allowed to rest for 20 min until it had clotted, at which time 1 ml of PBS was added. Supernatants were collected at the same points (15 min, 1 h and Days 1, 2, 4, 6, 8, 10, 12, 14).

#### HGF viability and proliferation assays

The effects of 3D bioprinted constructs on cellular viability were assessed using a fluorescent live/dead staining kit based on provided protocols (KeyGEN Bio TECH, KGAF001, China). Briefly, a 10-ml staining solution containing 8 µM of propidium iodide (PI) and 2 µM of calcein-AM was prepared. Scaffolds were stained for 30 min with this solution, washed three times with PBS, and then imaged via fluorescence microscopy, with viable cells staining green (calcein-AM, 490 nm) and dead cells staining red (PI, 535 nm). The sample imaging (*n* = 3) was performed on Days 1, 4 and 7, with numbers of live and dead cells being quantified using the ImageJ software. The cell viability was quantified by dividing live cell numbers by the total number of cells. A CCK-8 assay (LK815, Japan) was conducted based on provided protocols to assess HGF proliferation within 3D printed constructs on Days 1, 7 and 14. Briefly, samples were incubated with the CCK-8 reagent at these time points, after which the absorbance at 450 nm was assessed via microplate reader (BioTek ELX800, VT, USA).

#### qPCR

The gene expression in cells cultured for 14 days on 3D bioprinted constructs in individual groups (*n* = 4) was assessed via qPCR. Briefly, HGF-laden constructs were soaked for 5 min in 100 mM sodium citrate with gentle agitation to promote the de-cross-linking. Decapsulated cells were collected via centrifugation for 5 min at 1000 rpm. RNA was then extracted with an Aurum™ Total RNA Mini Kit (BioRad) based on provided directions, after which cDNA was prepared with a PrimeScript™ RT reagent kit (Takara, Japan). All qPCR analyses were conducted using the Faststart universal SYBR Green Master Mix (Rox) (Roche, Germany, 04913850001) and primers specific for Collagen I, Collagen III and Fibronectin synthesized by BGI (Table 3). Samples were analyzed in triplicate, with GAPDH being used as a normalization control.

**Table 3.** Primer sequences of RT-qPCR

Genes	Sequences
Collagen I	Forward: 5′-CGACAGCAGCCGCATCTT-3′
	Reverse: 5′-CCAATACGACCAAATCCGTTG-3′
Collagen III	Forward: 5′-CGCCCTCCTAATGGTCAAGG-3′
	Reverse: 5′-TTCTGAGGACCAGTAGGGCA-3′
Fibronectin	Forward: 5′-CCGCCGAATGTAGGACAAGAA-3′
	Reverse: 5′-CTGTCAGAGTGGCACTGGTA-3′

#### *In vivo* biocompatibility analyses

The biocompatibility of 3D bioprinted constructs containing cells was assessed *in vivo* by subcutaneously implanting HGF-laden control (AG) or experimental (50% iPAG) samples into the backs of nude mice. Briefly, 8-week-old male BALB/c nude mice (Charles River; 25 ± 1 g) were randomly assigned to the experimental or control groups and then intraperitoneally injected with 4% chloral hydrate for anesthetization. Mice were disinfected and draped, and then a ∼2.0 cm linear incision was made on the back of each animal. The 3D bioprinted constructs (10 mm × 10 mm × 2 mm) were subcutaneously implanted under a bluntly separated skin flap, after which absorbable sutures were used to close the wound. Nude mice were euthanized via over-anesthetization at 1, 2, 4 or 8 weeks post-implantation, at which time the skin flap from the implantation site was harvested for histological analyses. The Laboratory Animal Welfare Ethics Branch of Peking University Biomedical Ethics Committee approved all animal studies (LA2021039).

#### Histological and histocompatibility analyses

Collected samples were fixed for 24 h using 4% paraformaldehyde, dehydrated, paraffin-embedded, cut perpendicularly to the flap and then used to prepare 5 μm-thick sections that were subjected to hematoxylin/eosin (H&E) and Masson’s trichrome (MT) staining (*n* = 12/group). Samples were also used for immunohistochemical (IHC) staining with anti-rat CD31 and anti-rat F4/80 as markers for vascular endothelial and macrophage populations. Differences among groups were compared in a semi-quantitative fashion after imaging with a light microscope (Olympus, Japan). ImageJ (NIH, MD, USA) was used to quantify numbers of CD31+ blood vessels.

### Statistical analysis

Data were compared using SPSS 20.0 at a 95% confidence level using one-way ANOVAs with Tukey’s *posthoc* test. Data are given as means ± standard deviation. *P* < 0.05 was the threshold of significance.

## Results and discussion

### iPAG bioink preparation and characterization

#### iPAG bioink rheological evaluation

**Figure 3.**
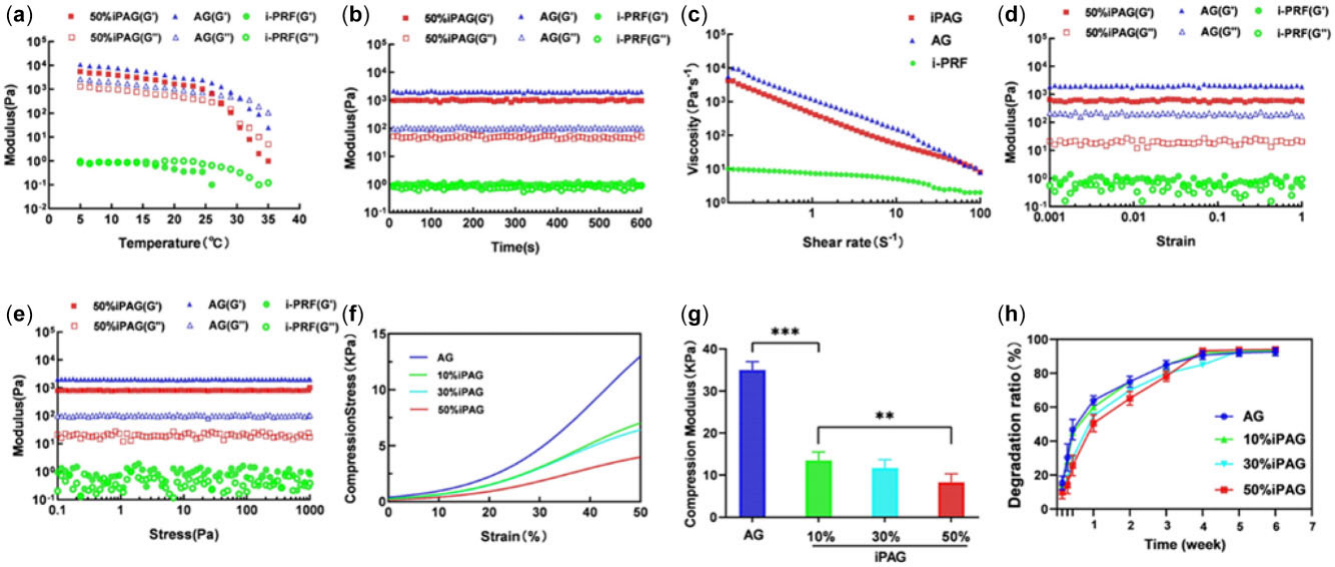
Rheological behavior and mechanical property of iPAG bioink in comparison to i-PRF and AG. (**a**) The modulus–temperature curve. (**b**) The modulus–time curve. (**c**) The viscosity–shear rate curve. (**d**) The modulus–strain curve. (**e**) The modulus–pressure curve. (**f**) The strain–stress curve. (**g**) The compression modulus of bioinks. (**h**) *In vitro* degradation curves of cell-free iPAG and AG constructs (**P < 0.01; ***P < 0.001)

Optimal bioink preparations should exhibit rheological properties consistent with their intended use. We therefore assessed the oscillatory and rotational rheological characteristics of cell-free iPAG bioink as a means of gauging its amenability to printing. The modulus–temperature curve (Fig. 3a) revealed that the sol–gel transition point for the bioink samples in each group was dependent on the temperature at which the storage modulus (G') exceeded the loss modulus (G″). When the temperature was reduced from 37°C to 5°C, the sol–gel critical temperature for the AG group was proximately 32°C. As the i-PRF proportion rose, this critical temperature gradually fell to 27.5°C in the 50% iPAG group. All subsequent rheological testing was then performed at 20°C. Modulus–time curves (Fig. 3b) indicated that the iPAG bioink exhibited loss and storage modulus values that remained relatively stable for over 600 s, consistent with a lack of substantial structural reorganization within the bioink during this period. The modulus–deformation curve (Fig. 3d) and the modulus–pressure curve (Fig. 3e) further revealed this iPAG bioink to exhibit a large linear viscoelastic region, consistent with its ability to remain stable even when subject to significant deformation. The viscosity–shear rate curve (Fig. 3c) revealed i-PRF to exhibit shear-thinning properties characterized by a drop in iPAG bioink viscosity with increasing shear rate. Fibrinogen, which is present at high levels in i-PRF, can form a polymerized fibrin network when in contact with calcium and/or thrombin. Such a cross-linked fibrin network is not suited to extrusion-based printing given that the resultant structure would not be robust [26], in accord with our findings. It is thus essential that platelet-enriched bioproducts can be combined with other materials when preparing a composite bioink such that the resultant samples exhibit rheological properties amenable to printing. Li *et al*. [27] designed a silk fibroin bioink containing PRP that was utilized to regenerate cartilage. Similarly, Irmak *et al*. [28] reported the development of a PRP-based bioink for use in cartilaginous tissue engineering in which Gel-MA was used to reinforce the PRP. Herein, AG was incorporated to enhance the extrusion performance of our bioink preparations. Relative to i-PRF, this composite iPAG bioink exhibited significantly more favorable rheological properties, performing similarly to AG such that it could be smoothly extruded to form printed layers that maintained the shape in which they were printed.

#### Compression modulus analyses

Understanding the mechanical properties of scaffolds used for tissue engineering is vital to mimic the native tissue more effectively, thus improving the ability of cells to attach and proliferate [29]. As such, we next assessed the compression modulus values for the prepared cell-free bioink (Fig. 3f and g). The AG bioink exhibited the highest compression modulus (35 ± 2 kPa), while this value fell as the i-PRF proportion within iPAG bioink samples rose, from 13.5 ± 2.2 kPa in the 10% iPAG group to 11.7 ± 1.6 kPa in the 30% iPAG group and 8.3 ± 0.5 kPa in the 50% iPAG group. The bioink mechanical stiffness is dependent upon the intensity of cross-linking [30]. The alginate concentration was the primary determinant of the degree of cross-linking in our study, with Ca^2+^ being employed to achieve such cross-linking. As the proportion of i-PRF increased, the concentration of alginate in the resultant bioink decreased, resulting in looser Ca^2+^-alginate cross-linking and lower compression modulus values. There was no other chemical crosslinking substance. Thus, i-PRF has a dilutive effect on the preparation of the iPAG bioink. The mechanical properties of these iPAG bioink samples fit well with the range of elasticity values observed for the oral soft tissue, which, like native skin, exhibits elasticity ranging from 10 to 100 kPa [31, 32].

#### *In vitro* degradation test

To be well-suited to use in the context of tissue regeneration, an effective biocompatible platform needs to avoid undergoing excess degradation at a rate that outpaces the regenerative process. Analyses of the longitudinal weight loss ratios of our prepared bioinks suggested that i-PRF incorporation did not impact the degradation rates of the cell-free scaffolds (Fig. 3h). This may be because i-PRF is primarily composed of fibrin, which degrades at a rate similar to gelatin. These scaffolds exhibited degradation rates that were significantly faster during the first week, but such degradation could be extended to 6 weeks, aligning well with the rate at which the oral soft tissue regeneration occurs.

### Preparation and characterization of 3D bioprinted constructs

In light of the above rheological data, the 3D printer and platform were set to an ambient temperature of 10°C such that the bioink was deposited in the form of thin filaments that maintained their shape in 3D printed cell-laden lattice structures (Fig. 2e and f). The prepared iPAG bioink was thus able to retain its shape when applied in a multi-layered format, making it potentially well-suited to use in the fabrication of replacement oral soft tissue for the repair of appropriate defects.

#### Surface morphology analyses

**Figure 4.**
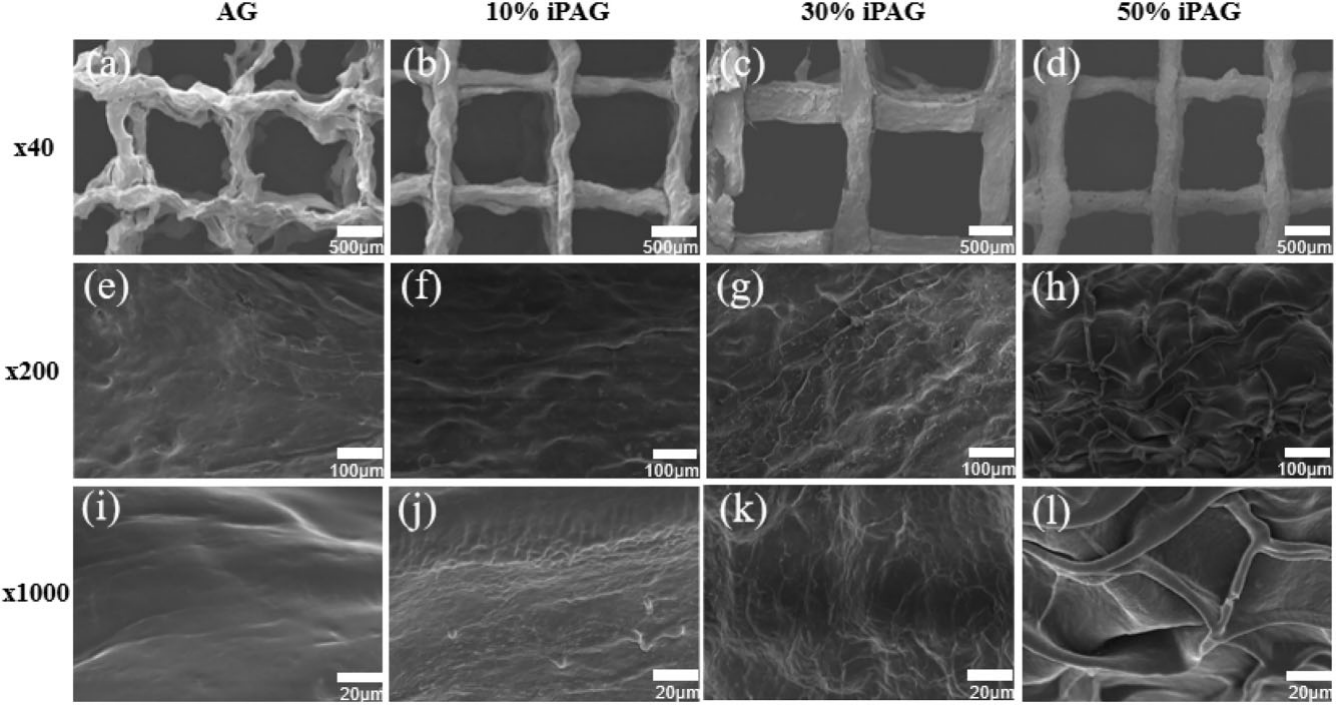
SEM images of (**a**, **e**, **i**) AG; (**b**, **f**, **j**) 10% iPAG; (**c**, **g**, **k**) 30% iPAG and (**d**, **h**, **l**) 50% iPAG constructs

The prepared freeze-dried 3D printed cell-free scaffolds exhibited grid-like surface characteristics, with clear differences among groups being evident at higher levels of magnification (Fig. 4). While AG samples exhibited a smooth surface, strip-shaped fibers covered the surfaces of samples in the iPAG groups, with these fibers becoming denser as the proportion of i-PRF in the prepared bioink rose. These fibers exhibited an interwoven networked structure in the 50% iPAG group, potentially owing to the formation of fibrin fibers derived from the fibrinogen in i-PRF. These findings are consistent with prior reports regarding the 3D fibrin matrix of i-PRF [14]. This fibrin can serve as a scaffold to support cell adherence in the context of tissue regeneration, in addition to carrying growth factors and thereby supporting their controlled, sustained release in a which is conducive to prolonged regenerative bioactivity [33].

#### *In vitro* growth factor release analyses

**Figure 5.**
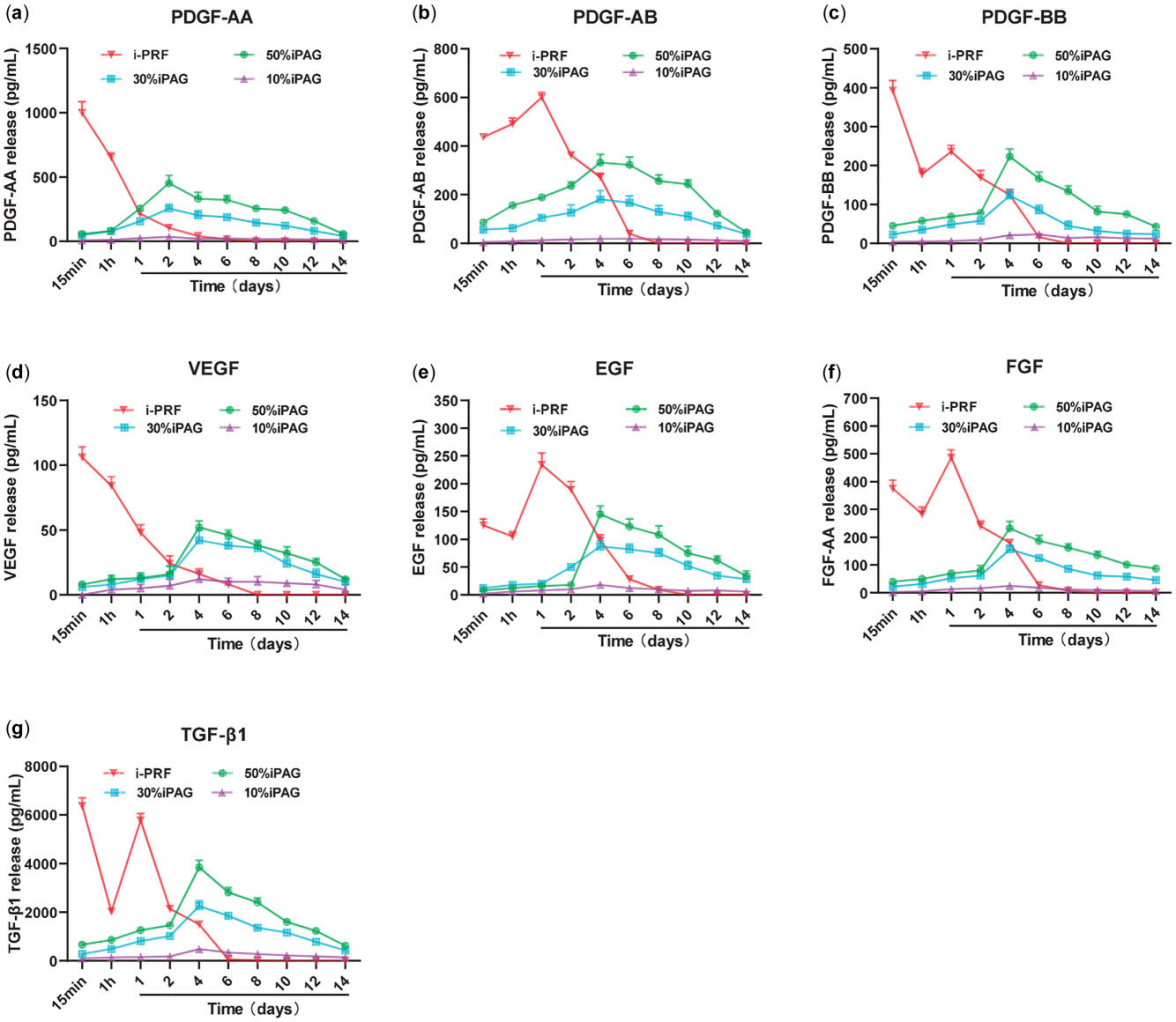
ELISA protein quantification for 10%, 30% and 50% iPAG constructs and i-PRF at each time point of (**a**) PDGF-AA; (**b**) PDGF-AB; (**c**) PDGF-BB; (**d**) VEGF; (**e**) EGF; (**f**) FGF and (**g**) TGF-β1 over a 14-day period

Different growth factors have been shown to promote the migratory, proliferative and differentiation activity of cells in both additive and synergistic manners *in vitro* and *in vivo*, thereby promoting enhanced healing activity [34]. As a cost-effective source of these growth factors, i-PRF can be readily prepared from autologous samples in a personalized manner. An ELISA approach was used to measure the release of PDGF-AA, PDGF-AB, PDGF-BB, VEGF, EGF, FGF and TGF-β1 from i-PRF-containing samples. In i-PRF samples (Fig. 5), we found that these growth factors were rapidly released in a burst over the initial 2 days, after which their release was far more gradual. The cumulative PDGF-AA, PDGF-AB and TGF-β1 release levels were far higher than those of other growth factors (>1000 to 10 000 pg/ml), with TGF-β1 exhibiting maximal release in this assay context. Kobayashi *et al*. previously reported the highest growth factor released from platelet concentrates was PDGF-AA followed by PDGF-BB, TGF-β1, VEGF and PDGF-AB. Interestingly, in the above study, PRF demonstrated the ability to release high levels of growth factors up to a 10-day period and different formulation of PRF had different release profiles, which may contribute to different cell content in platelet concentrates [16].

A cumulative release curve was used to monitor the release of these same growth factors from 10%, 30% and 50% iPAG bioink preparations over a 2-week period (Supplementary Fig. S1). Consistent with other previously reported hydrogels, these bioink samples exhibited an initial rapid release phase followed by more gradual but sustained release over the remainder of the 2-week period. Notably, such growth factor release was far more gradual than that from the i-PRF samples even after the culture for 2 weeks, pronounced that the growth factor release was still detectable. The concentrations of released growth factors rose in proportion to the amount of i-PRF within the bioink samples, slower compared to i-PRF. This suggests that bioink samples generated through the mixture of i-PRF and AG can promote the prolonged and sustained growth factor release. Consistently, Liu *et al*. [35] previously reported the sustained release of growth factors from hyaluronic acid (HA)/PRP hydrogels over a 14-day period, while Daikuara *et al*. [36] shown the prolonged growth factor release from platelet lysates incorporated into a gelatin methacryloyl (GelMA) bioink. In line with these previous reports, our results confirmed the ability of a platelet derivative/hybrid polymer to promote the prolonged growth factor delivery in a manner conducive to the retention of bioactivity. Given the direct incorporation of unmodified i-PRF into these bioink formulations, such growth factor retention is likely attributable to the mechanical properties of the bioink polymer and to electrostatic interactions between growth factors and the gelatin or fibrin network matrix.

#### HGF viability and proliferation analyses

**Figure 6.**
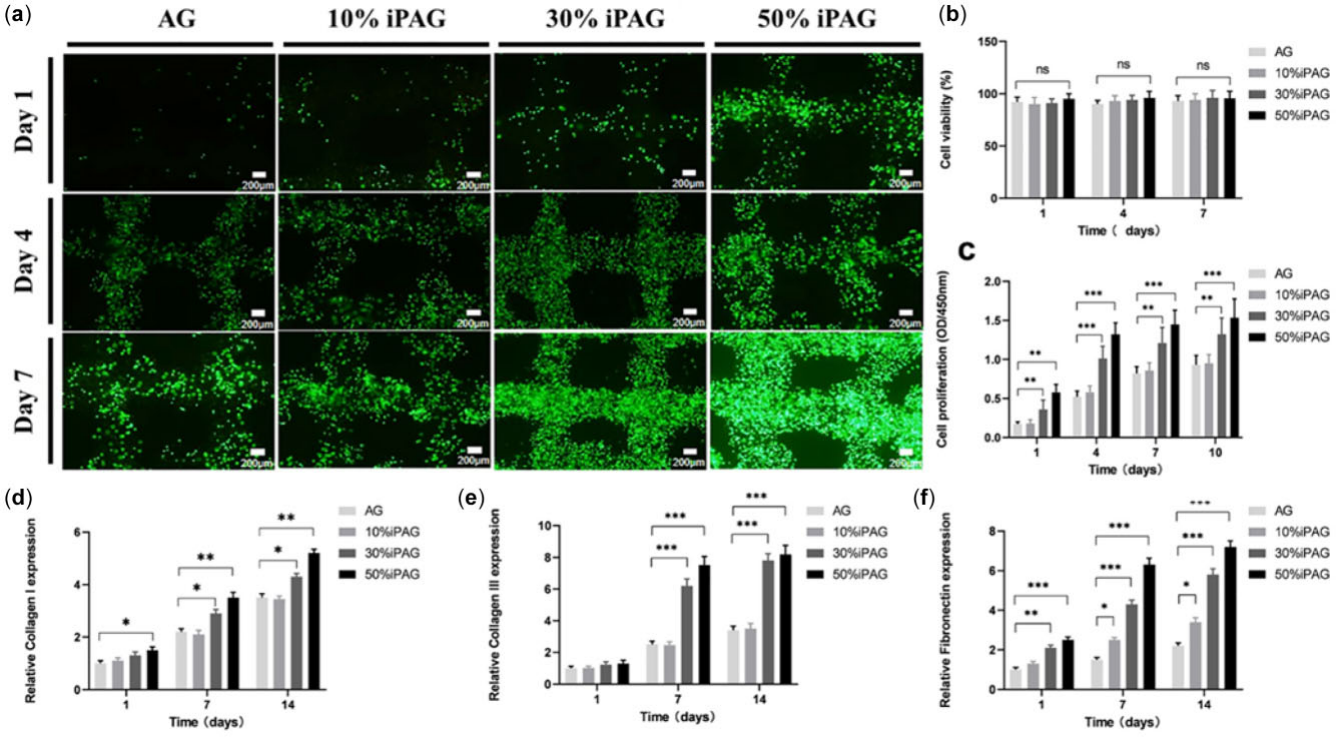
(**a**) Fluorescence images of live/dead stained samples on Days 1, 4 and 7. HGF cells were encapsulated within the bioinks at a concentration of 1 × 10^6^ cells/ml. Cytocompatibility test results with HGF cells. (**b**) Calculated cell viability of cultured cells in bioinks (*n* = 3). (**c**) CCK-8 assay results at 1, 4, 7 and 10 days of cultured HGF cells (*n* = 3). Relative expression of (**d**) Collagen I; (**e**) Collagen III and (**f**) fibronectin detected by qPCR in different groups on Days 1, 7 and 14 (*P < 0.05; **P < 0.01; ***P < 0.001; ns, no significance)

The efficacy of growth factor-based treatment as an approach to promoting tissue regeneration is highly dependent upon the preservation of the bioactivity of these factors throughout the fabrication and application process [37]. We thus explored the bioactivity of growth factors contained within our 3D printed iPAG constructs through *in vitro* assays conducted using HGFs. A live/dead staining assay was used to evaluate the HGF viability within a 7-day *in vitro* culture period (Fig. 6a), revealing high levels (≥96%) of viability (Fig. 6b) for the 3D printed HDF-iPAG within 24 h following the homogenous encapsulation of these cells within the prepared bioink and such viability was maintained for 7 days. Relative to Day 1, the live cell density on Day 7 in the bioink matrix rose markedly in the iPAG sample groups, with higher i-PRF concentrations being conducive to more robust proliferative activity. Conversely, cell proliferation was limited for AG scaffolds based on the CCK-8 assay data (Fig. 6c). The highest rates of proliferation were observed in the 50% iPAG group on Day 10, with an absorbance value nearly two times that in the AG control group, thus confirming the beneficial effects of growth factors on cellular viability and proliferation. Prior studies have confirmed the ability of i-PRF-derived growth factors to enhance cellular migration, proliferation and adhesion [19, 20]. Faramarzi *et al*. [38] reported that PRP combined with alginate was able to enhance mesenchymal stem cell migration and recruitment. The fibrin network within these preparations also provides many sites for cell anchorage that are conducive to the survival and adhesion of cells in the implantation site. Together, these results underscore the ability of the fabricated iPAG bioink to serve as a porous extracellular matrix (ECM) biomimetic network and source of blood-derived growth factors capable of influencing cellular survival and function.

#### Gene expression analyses

By producing ECM components such as Collagen I/III, elastin, HA and fibronectin, gingival fibroblasts support the integrity of the connective tissue within the oral environment. Collagens serve as the primary structural components within the gingival ECM, with type I/III collagen being particularly important in the context of oral soft tissue repair [39]. Fibronectin can further regulate granulation tissue neovascularization and the formation of a fibrillar collagen I/III network [40]. We thus next explore the impact of our prepared iPAG constructs on gingival fibroblast-mediated ECM deposition by assessing the expression of Collagen I/III and fibronectin via qPCR on Days 1, 7 and 14. This analysis revealed that Collagen I expression (Fig. 6d) differed between Days 1 and 14 in all groups, with Collagen I levels being significantly higher in the 30% and 50% iPAG groups as compared to the control and 10% iPAG groups on Day 14. There were no significant differences in Collagen III expression (Fig. 6e) between the AG and 10% iPAG groups on Days 1, 7 or 14, whereas its expression was significantly increased in the 30% and 50% iPAG groups on Days 7 and 14 relative to control and 10% iPAG samples. Similarly, the relative fibronectin expression (Fig. 6f) rose for the iPAG groups on Days 7 and 14. Overall, these results suggested that the iPAG-derived growth factors enhanced the production of ECM synthesized by gingival fibroblasts over a 2-week culture period. Both growth factors and the ECM are essential throughout the healing process, exhibiting bi-directional interactions such that the ECM can regulate growth factor signaling and binding, while growth factors can control the synthesis or degradation of the ECM through various pathways [41].

#### Histological analysis

**Figure 7.**
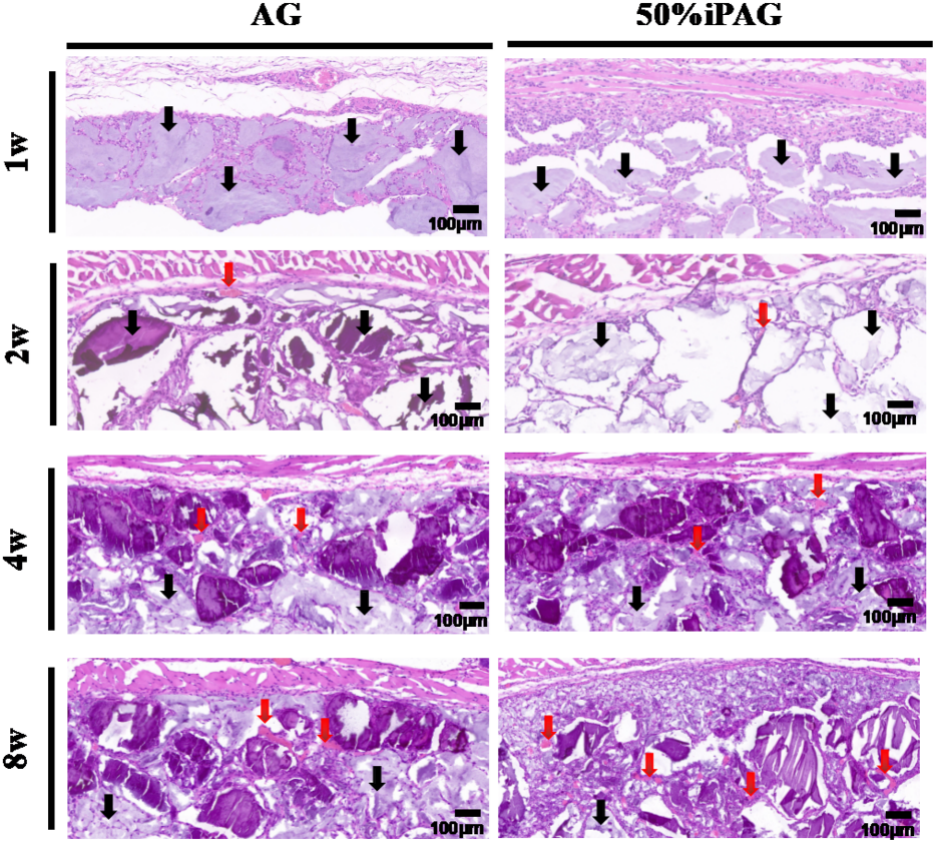
H&E staining after 1, 2, 4 and 8 weeks of implantation. The constructs were indicated by the black arrow. Blood vessels were indicated by the red arrow

**Figure 8.**
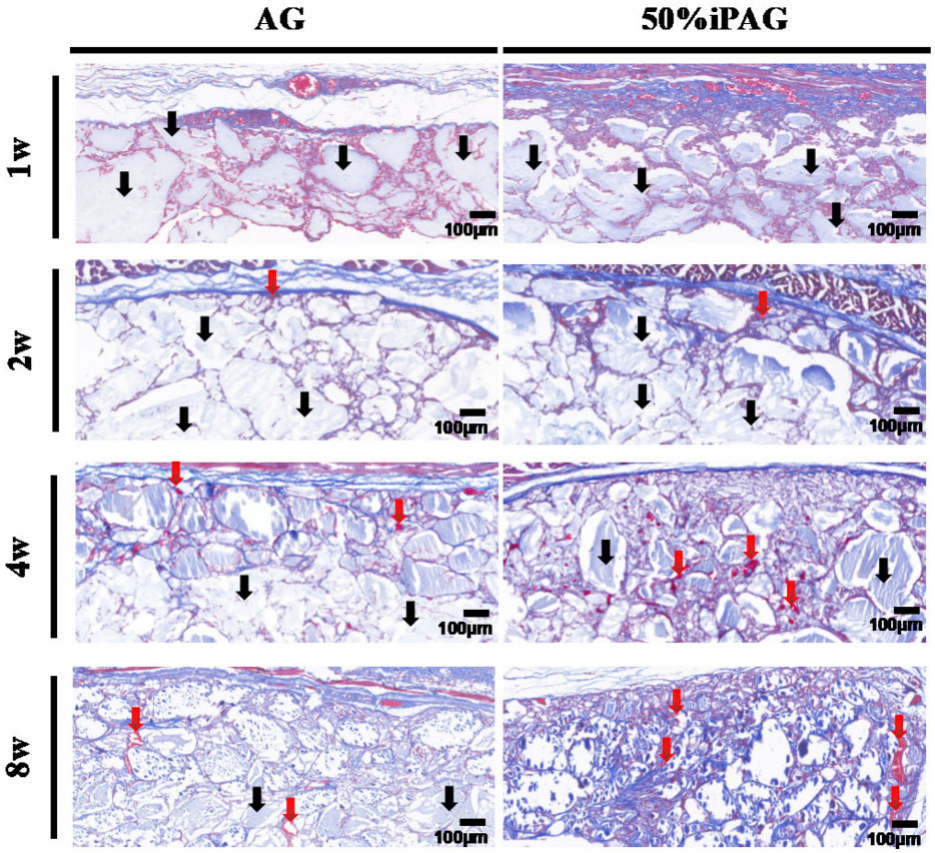
MT staining after 1, 2, 4 and 8 weeks of implantation. The constructs were indicated by the black arrow. Blood vessels were indicated by the red arrow

The results of H&E staining (Fig. 7) revealed that both 3D bioprinted constructs were visible within the subcutaneous layer without any marked concomitant inflammation. Over time, the development of new blood vessels in the implant site was more clearly evident in the iPAG group relative to the AG group. At 4 weeks post-implantation, these constructs were not fully degraded. Host tissue infiltration occurred more rapidly in the iPAG group relative to the AG group such that at 4 weeks post-implantation, 70% and 50% surrounding tissue infiltration were evident in these two respective groups. MT staining (Fig. 8) was additionally conducted to evaluate regenerated collagen and fibrosis proximal to the implanted constructs. Regenerated collagen was denser and more extensively distributed in the iPAG group relative to the AG group, while no severe fibrosis was observed in either group. Abd El Raouf *et al*. [42] have similarly reported the ability of autologous i-PRF to facilitate the robust and rapid repair of cartilaginous tissue likely owing to the high leukocyte, platelet, and growth factor concentrations therein conducive to the enhancement of chondroblast and BMSC proliferation and migration during this repair process. Lu *et al*. [43] revealed excellent biocompatibility of i-PRF, which could significantly promote human dermal papilla cell proliferation, migration, and trichogenic inductivity. These results, together with these prior data, thus suggest that i-PRF can more readily promote the subcutaneous soft tissue regeneration in a stable and evenly distributed manner owing to the ability of this 3D bioprinted construct to promote enhanced cell recruitment.

#### Histocompatibility analysis

**Figure 9.**
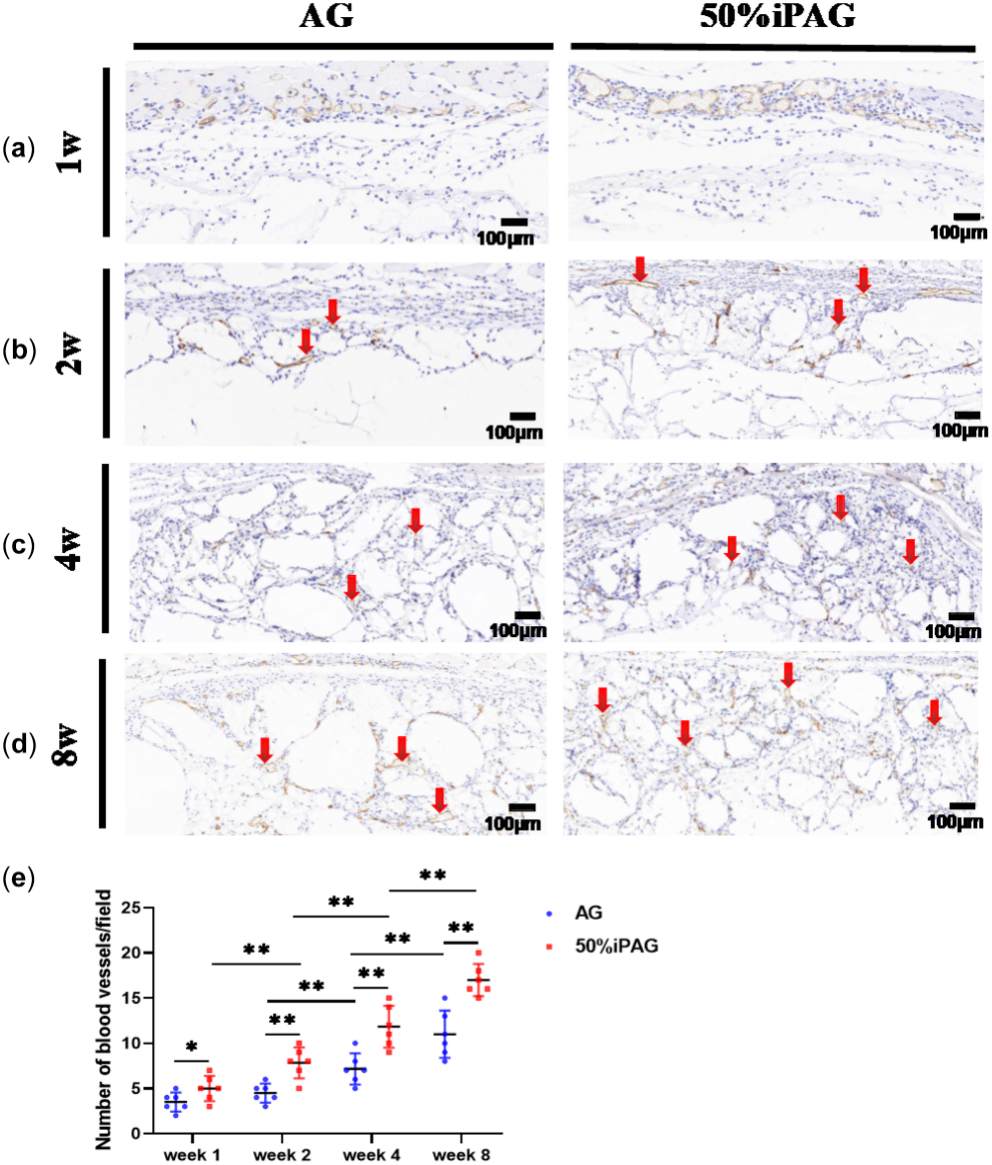
CD31 immunostaining after 1 (**a**), 2 (**b**), 4 (**c**) and 8 (**d**) weeks of implantation. Blood vessels were indicated by the red arrow. (**e**) Semiquantitative analysis of vascular distribution. The number of new blood vessels in the iPAG group was significantly higher than that of AG group. (*P < 0.05; **P < 0.01)

Angiogenesis is integral to the wound healing process, with oxygen, minerals and essential nutrients all being derived from the local vasculature to support regenerative activities [44]. To assess such angiogenic activity surrounding the implanted constructs, we conducted IHC staining for the vascular endothelial cell marker CD31 (Fig. 9). At 2 weeks post-implantation, an extensive network of new blood vessels was observed surrounding constructs in the iPAG and AG groups, although the number of such vessels was significantly higher in the iPAG group relative to the AG group. About 20% increase in new blood vessel formation were observed in the AG group at 4 weeks post-implantation, whereas a roughly 50% increase in the number of new blood vessels in the iPAG group was observed at this time point relative to at 2 weeks post-implantation (Fig. 9e). At 8 weeks post-implantation, these new blood vessels were densely distributed across the implanted construct in the iPAG group and exhibited a significant increase in average vessel diameter. These results were thus consistent with the ability of i-PRF to promote local angiogenic activity, consistent with prior data from Yuan *et al*. [45], which demonstrated the enhanced early angiogenesis following i-PRF application into an extracted socket. These results are also consistent with the high density of autologous growth factors with known angiogenic activity present within i-PRF, including both PDGF and VEGF [46], particularly given our *in vitro* evidence indicating that iPAG bioink preparations were able to better promote the sustained growth factor release.

**Figure 10.**
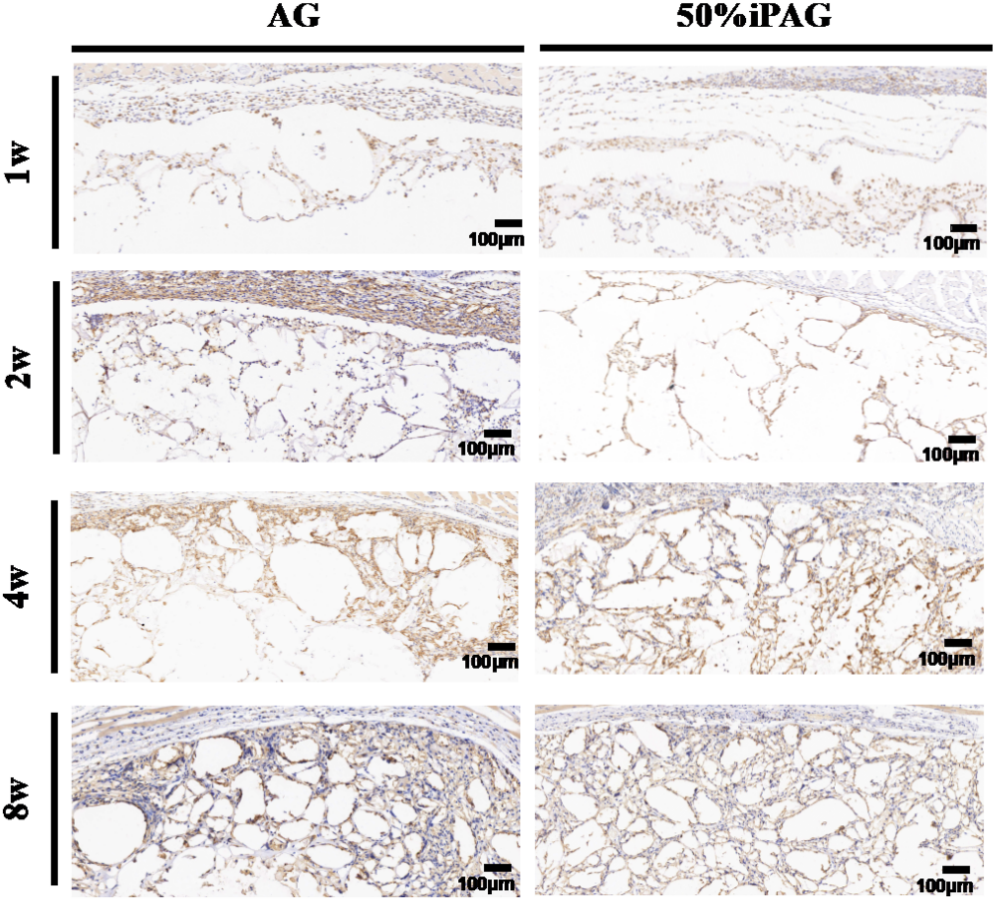
F4/80 immunostaining after 1, 2, 4 and 8 weeks of implantation

The introduction of grafted tissue into a healing wound can significantly prolong the local inflammatory and proliferative phases of the regenerative process. By providing sustained local stimulation, grafts can ultimately promote unintended chronic inflammatory activity conducive to connective tissue build-up and fibrosis that may ultimately result in graft rejection, dysfunction or destruction. Inhibiting such inflammatory activity is thus essential to ensure maximal biocompatibility. To explore such biocompatibility in our assay system, we explored macrophage infiltration proximal to the site of inflammation using the macrophage marker F4/80 (Fig. 10), as these cells are commonly associated with the activation of an inflammatory response. We observed some level of inflammation in both groups from 1 to 2 weeks post-implantation, consistent with a normal immune response to improve the local healing. While limited macrophage infiltration was still evident in the AG group at weeks 4–8 post-implantation, no such inflammation was evident in the iPAG group. In a prior report exploring the anti-inflammatory effects of i-PRF on macrophages and dendritic cells, researchers found such treatment was sufficient to reduce proinflammatory M1 macrophage differentiation and dendritic cell activation proximal to a muscle tissue defect, in which a bacterial suspension had been applied [47]. Yuan *et al*. [45]. further found that i-PRF-derived anti-inflammatory factors were similarly able to suppress proinflammatory M1 macrophage phenotypes. In summary, the iPAG constructs developed herein were highly biocompatible while exerting robust anti-inflammatory and angiogenic activity.

## Conclusions

In conclusion, we have herein presented a novel approach to facilitating personalized extruded bioink-based i-PRF treatment strategy, which can facilitate the sustained release of autologous growth factors to promote oral soft tissue regeneration. This bioink, composed of a mixture of alginate, gelatin and i-PRF, exhibited mechanical and rheological properties for applying in 3D bioprinting and possessed excellent biocompatibility *in vitro* and *in vivo*. Most notably, when deployed subcutaneously *in vivo*, this bioink was able to simultaneously suppress inflammation while promoting angiogenesis. These results provide a foundation for the fabrication of more complex biological structures using a 3D bioprinting approach, enabling the better mimicking of native organs and tissues in a manner that is conducive to homogenous and multi-layered biofactor distribution.

## Supplementary data

Supplementary data are available at *REGBIO* online.

## Funding

This work was supported by the National Key Research and Development Program of China (2017YFA0701302, PKUSS20200113).

*Conflict of interest statement.* None declared.

## Supplementary Material

rbac021_Supplementary_Data
