# Supplementary figures and images for: Utilizing 3D bioprinted platelet-rich fibrin-based materials to promote the regeneration of oral soft tissue
Source: Regen Biomater. 2022 Apr 13;9:rbac021. doi: 10.1093/rb/rbac021 (PMC9086746; doi:10.1093/rb/rbac021)

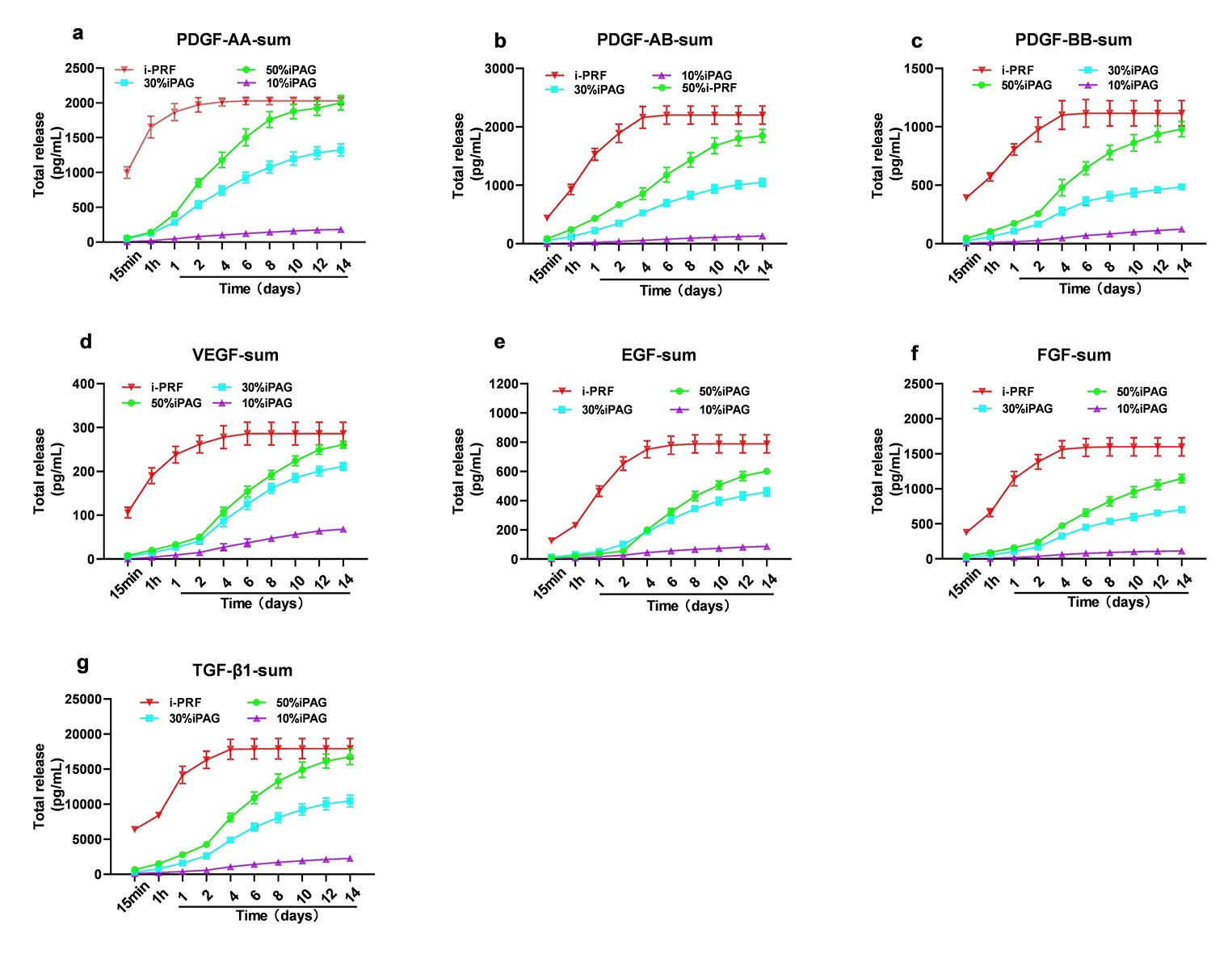

Supplement: rbac021_Supplementary_Data [file rbac021_supplementary_data.jpeg]
